# Subarachnoid hemorrhage secondary to a ruptured middle cerebral aneurysm in a patient with osteogenesis imperfecta: a case report

**DOI:** 10.1186/1471-2377-14-150

**Published:** 2014-07-23

**Authors:** Toshio Hirohata, Satoru Miyawaki, Akiko Mizutani, Takayuki Iwakami, So Yamada, Hajime Nishido, Yasutaka Suzuki, Shinya Miyamoto, Katsumi Hoya, Mineko Murakami, Akira Matsuno

**Affiliations:** 1 Department of Neurosurgery, Teikyo University Chiba Medical Center, 3426-3 Anesaki, Ichihara City, Chiba 299-0111, Japan; 2 Department of Neurosurgery, The University of Tokyo, 7-3-1 Hongo, Bunkyo-ku, Tokyo 113-8655, Japan; 3 Teikyo Heisei University, 2-51-4 Higashi-Ikebukuro, Toshima-ku, Tokyo 170-8445, Japan

**Keywords:** Osteogenesis imperfecta, Subarachnoid hemorrhage, Intracranial aneurysm, Type 1 collagen

## Abstract

**Background:**

Osteogenesis imperfecta (OI) is a heterogeneous group of inherited disorders that occur owing to the abnormalities in type 1 collagen, and is characterized by increased bone fragility and other extraskeletal manifestations. We report the case of a patient who was diagnosed with OI following subarachnoid hemorrhage (SAH) secondary to a ruptured saccular intracranial aneurysm (IA).

**Case Presentation:**

A 37-year-old woman was referred to our hospital because of sudden headache and vomiting. She was diagnosed with SAH (World Federation of Neurosurgical Society grade 2) owing to an aneurysm of the middle cerebral artery. She then underwent surgical clipping of the aneurysm successfully. She had blue sclerae, a history of several fractures of the extremities, and a family history of bone fragility and blue sclerae in her son. According to these findings, she was diagnosed with OI type 1. We performed genetic analysis for a single nucleotide G/C polymorphism (SNP) of exon 28 of the gene encoding for alpha-2 polypeptide of collagen 1, which is a potential risk factor for IA. However, this SNP was not detected in this patient or in five normal control subjects. Other genetic analyses did not reveal any mutations of the COL1A1 or COL1A2 gene. The cerebrovascular system is less frequently involved in OI. OI is associated with increased vascular weakness owing to collagen deficiency in and around the blood vessels. SAH secondary to a ruptured IA with OI has been reported in only six cases.

**Conclusion:**

The patient followed a good clinical course after surgery. It remains controversial whether IAs are caused by OI or IAs are coincidentally complicated with OI.

## Background

Osteogenesis imperfecta (OI) is a heritable connective tissue disorder, caused by abnormalities in type 1 collagen, and is characterized by bone fragility and other extraskeletal features, including hearing loss, blue sclerae, dentinongenesis imperfecta and hyperlaxity of the ligaments and skin [1]. Patients with OI show a wide range of clinical severities from being nearly asymptomatic, with individuals leading a normal life, to being severe, with individuals showing bone and connective tissue deformities resulting in perinatal death [2].

OI can cause diverse vascular complications such as aortic and carotid artery dissection, cardiac valvulopathy and coronary artery aneurysms [3-7]. It has been reported that the collagen type 1 alpha-2 gene (*COL1A2*) may predispose patients to intracranial aneurysms (IAs) [8], and that the cerebrovascular system is less frequently involved in OI. We report the case of a patient in whom OI was diagnosed following subarachnoid hemorrhage (SAH) secondary to a ruptured intracranial saccular aneurysm.

## Case Presentation

**Figure 1:**
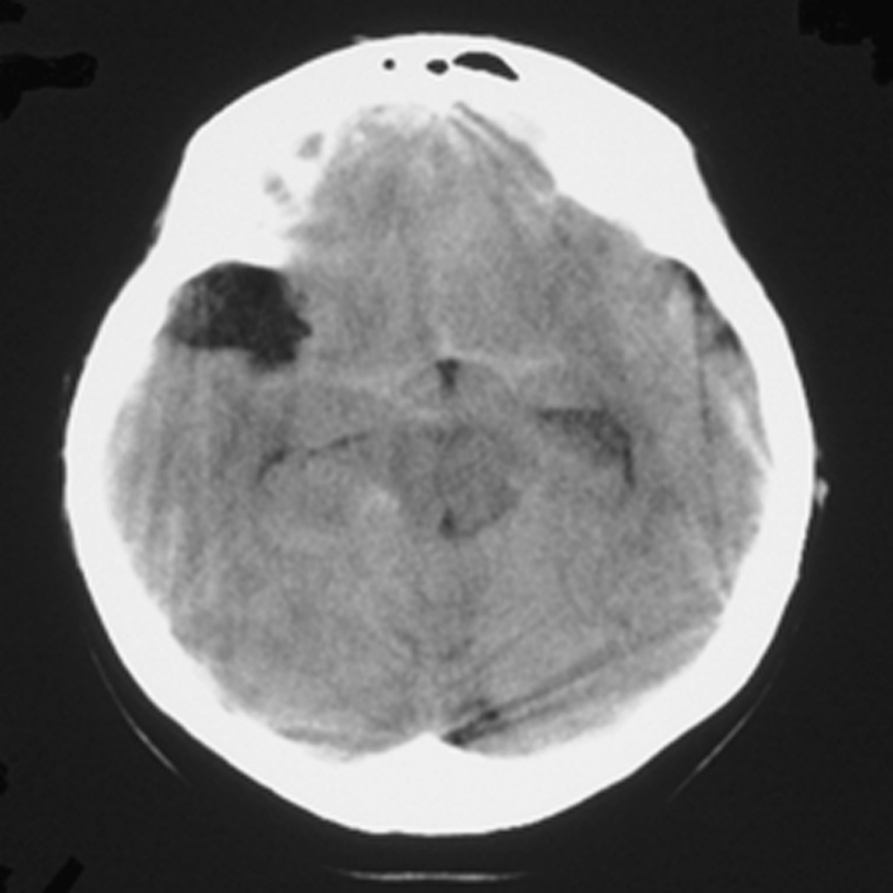
Computed tomography scan of the brain on arrival at the hospital, indicating diffuse subarachnoid hemorrhage and an arachnoid cyst at the right middle fossa.

**Figure 2:**
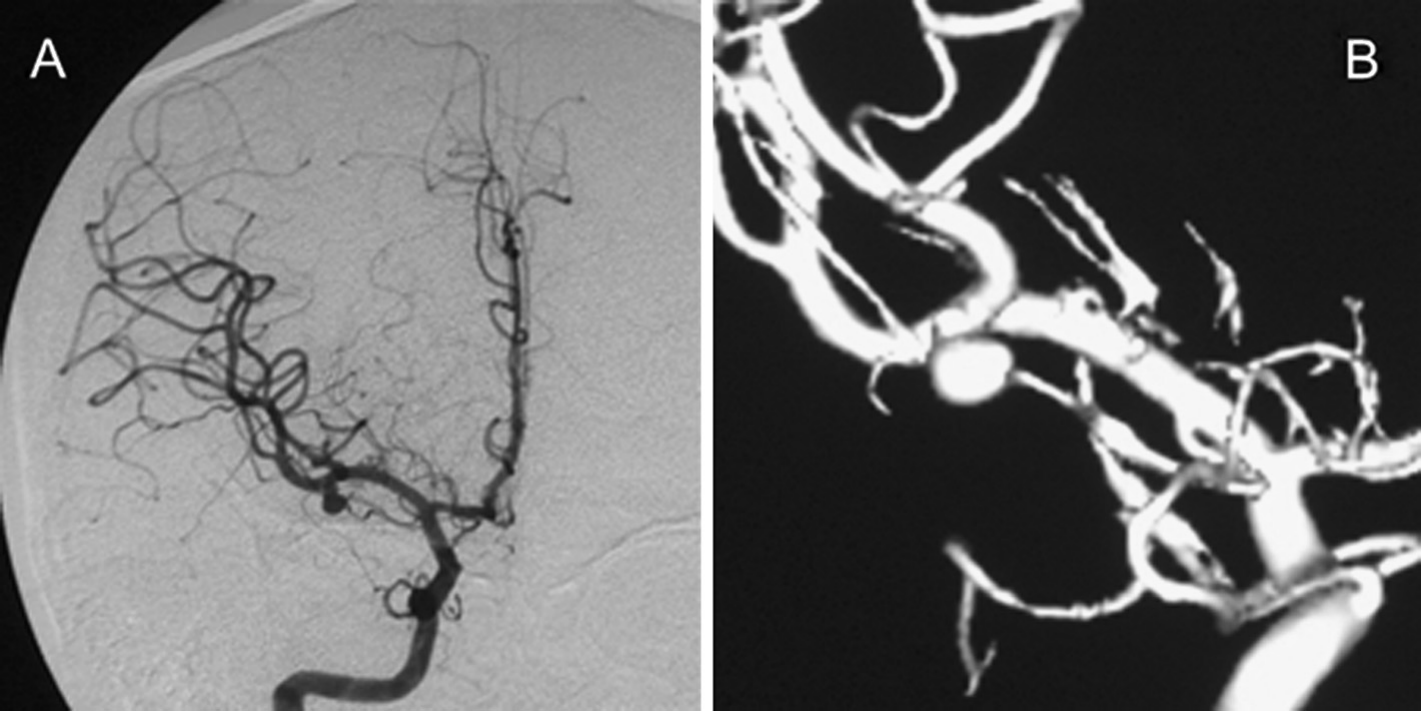
**Preoperative cerebral digital subtraction angiography showing a right middle cerebral artery aneurysm (5 mm in diameter). (A)** Right internal carotid angiography in the anteroposterior direction. **(B)** Three-dimensional angiography.

**Figure 3:**
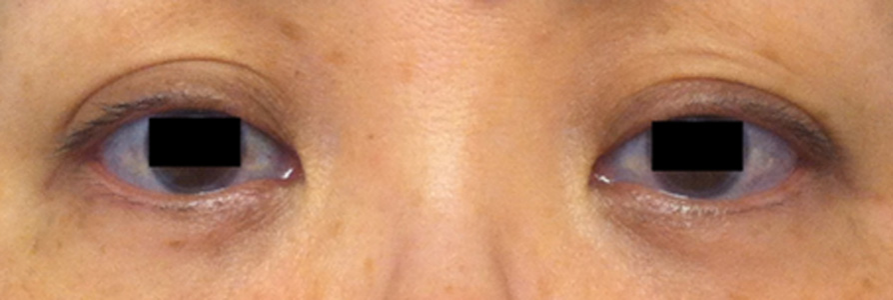
Photograph of the patient’s eyes, showing typical blue sclerae.

A 37-year-old woman was referred to our hospital because of sudden headache and vomiting. She had no family history of aneurysms. Neurologic examination revealed slight disturbance of consciousness (Glasgow Coma Scale score, 14) and neck stiffness without any focal deficit. Brain computed tomography (CT) showed diffuse SAH (Fisher stage 3) and an arachnoid cyst of the right middle fossa (Figure 1). Subsequent cerebral digital subtraction angiography (DSA) indicated a saccular aneurysm of the middle cerebral artery (Figure 2). Because of the ruptured IA, a diagnosis of SAH (World Federation of Neurosurgical Society grade 2) was made. The patient had blue sclerae (Figure 3), hypertension, and mitral regurgitation (New York Heart Association class 2), and had sustained repeated fractures of the extremities such as left elbow joint and left ankle joint prior to puberty. Her height was 158 cm (equal to the average height of Japanese women) with no major skeletal deformities. The patient’s son also had blue sclerae and a history of multiple fractures; however there was no history of bone fragility in her parents, two brothers or daughter. According to these findings, the diagnosis of OI type 1A was made during admission [9].

**Figure 4:**
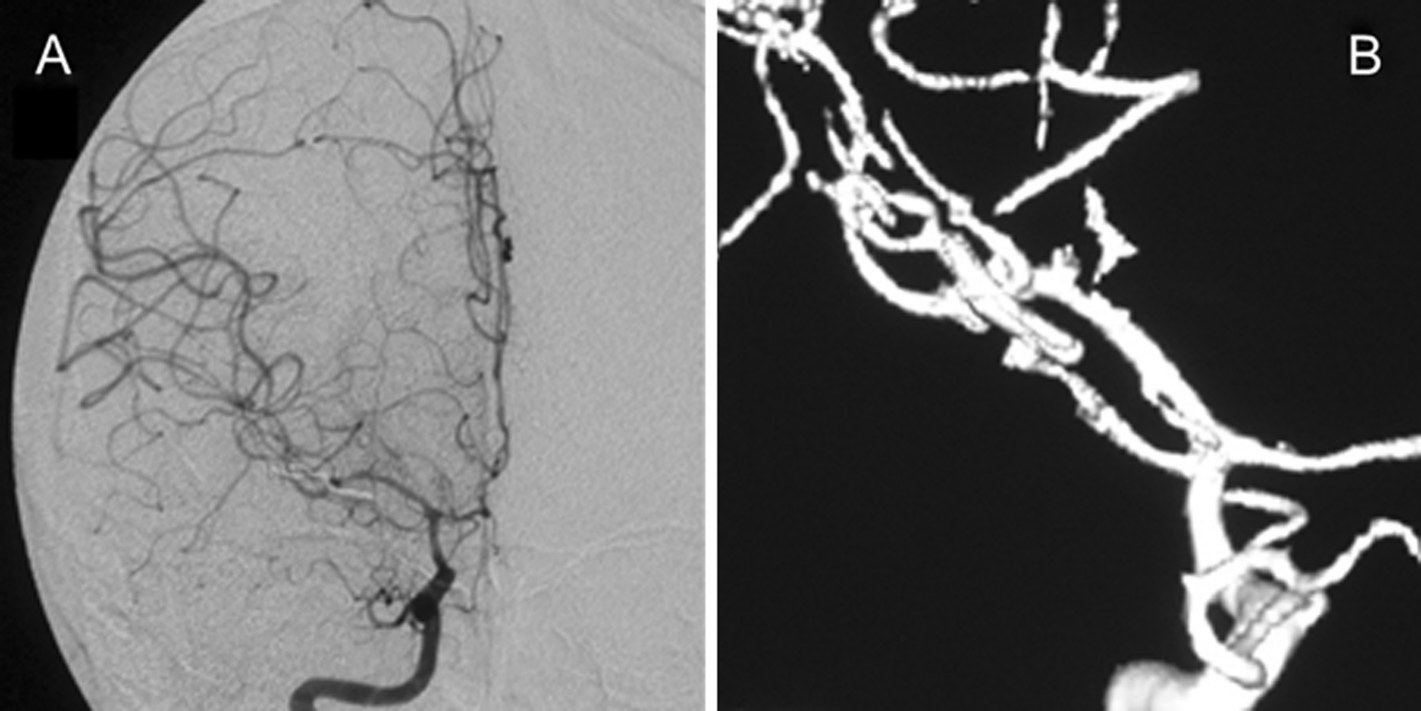
**Postoperative cerebral digital subtraction angiography showing a right middle cerebral artery aneurysm that has been successfully clipped. (A)** Right internal carotid angiography in the anteroposterior direction. **(B)** Three-dimensional angiography.

Surgical clipping of the aneurysm was performed, and the patient was asymptomatic after one week. Follow-up DSA demonstrated a completely clipped aneurysm (Figure 4). The patient underwent rehabilitation and was discharged from the hospital four weeks after admission.

The patient’s left femoral bone density was 0.752 g/cm^2^, which was in the Japanese female average. We also measured levels of serum bone metabolism markers, and they were within normal limits found in adult women; pyridinoline cross-linked carboxyterminal telopeptide of type I collagen, 1.1 ng/mL (normal range, <4.5); intact amino-terminal propeptide of type I procollagen, 16.6 μg/L (normal range, 14.9-68.8); tartrate-resistant acid phosphatase-5b, 147 mU/dL (normal range, 120–420); osteocalcin, 10 ng/mL (normal range, 2.5-13); and bone-specific alkaline phosphatase, 9.2 μg/L (normal range, 2.9-14.5).

**Figure 5:**
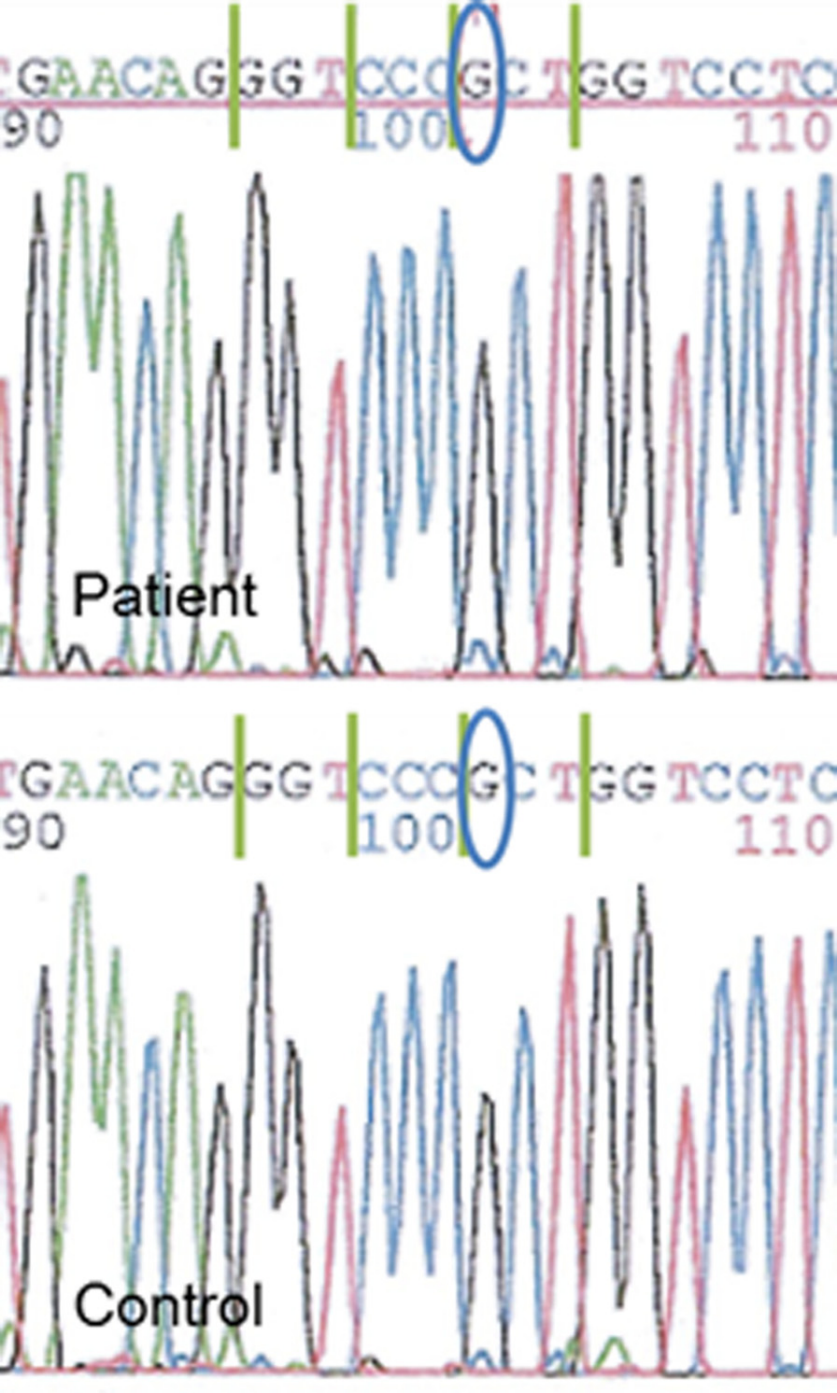
**Results for determining a single nucleotide G/C polymorphism (SNP) of exon 28 of the gene encoding for alpha-2 polypeptide of collagen 1 in the patient (upper panel) and one of normal control subjects (lower panel).** The SNP28 of COL1A2 was not detected in any of them.

We performed genetic analysis for a single nucleotide G/C polymorphism (SNP) of exon 28 of the *COL1A2* gene, which was reported as a potential risk factor for IAs [8]. However, this SNP was not detected in this patient or five normal control subjects (Figure 5). And no mutation of the *COL1A1* or *COL1A2* gene was detected with genetic analyses.

## Discussion

The diagnosis of OI is based mainly on clinical signs, symptoms, and positive family history [10]. Most patients with OI have a mutation of either the *COL1A1* or *COL1A2* gene, which are the two genes that code for alpha-1 and alpha-2 chains of type 1 collagen, respectively. More than 2,000 different type 1 collagen mutations have been identified in patients with OI and are listed in a database [11,12]. Type 1 collagen is the most abundant connective tissue in the vertebrate and is found in other tissues, including blood vessels [13]. Interstitial type 1 collagen was reported to be deposited on the surfaces of smooth muscle cells or along elastin filaments in the cerebrovascular wall, and thus, type 1 collagen might play a role in the rigidity and elasticity of the vascular wall. We did not identify any known mutation in *COL1A1* or *COL1A2* in our patient. However, OI was directly diagnosed in this patient because of the presence of bone fragility, extraskeletal features, including blue sclerae and mitral regurgitation, and a positive family history, despite her normal bone density and normal bone metabolism markers. The association between OI and an intracranial arachnoid cyst is also known [14], similarly as demonstrated in brain CT scan of this patient.

Yoneyama et al. [8] reported the association between the functional variant SNP28 of *COL1A2* and Japanese familial IA. However, in that study, SNP28 was observed in only 10.4% of total IAs (n = 260) and 5.5% of control subjects (n = 291). Although SNP28 was not detected in our patient and normal control subjects, we consider these results as acceptable regarding low prevalence of the SNP. Meanwhile, most of the vascular complications in patients with OI have been reported as artery dissections [3-7]. A few sporadic case reports described intracranial artery dissections complicated by OI [15,16].

To our knowledge, SAH secondary to ruptured IA in patients with OI has been reported in only six cases (Table 1) [17-22]. Three case reports have described patients with a saccular anterior communicating artery aneurysm [17-19], and another described a patient with a saccular aneurysm at a fenestrated basilar artery [20]; this is the only case report of SAH in a patient with OI in whom the SNP28 of the *COL1A2* gene was detected. The fifth report described a patient with a ruptured dissecting-type pseudoaneurysm at the right superior cerebellar artery [21]; this is the only case in which SAH secondary to an intracranial artery dissection was reported. The sixth and most recent report described a patient with a saccular aneurysm of the vertebral artery [22]. It is difficult to determine whether ruptured, in particular saccular, IAs are truly caused by OI or accidentally complicated by OI.

**Table 1:** Case reports of subarachnoid hemorrhage secondary to ruptured intracranial aneurysm with osteogenesis imperfecta

**Authors (year)**	**Age, Sex**	**Location**	**Shape**	**Supplement**
Okamura T, et al. (1995) [17]	33 F	A-com A	Saccular	VA fenestration
Narváez J, et al. (1996) [18]	22 F	A-com A	Saccular	
Havlik DM, et al. (2006) [19]	38 M	A-com A	Saccular	
Petruzzellis M, et al. (2007) [20]	44 M	VA union	Saccular	SNP28 of *COL1A2*: detected VA fenestration
Matouk CC, et al. (2011) [21]	49 M	SCA	Dissection	
Kaliaperumal C, et al. (2011) [22]	53 M	VA	Saccular	
Our case (2013)	37 F	MCA	Saccular	SNP28 of *COL1A2*: not detected

*SAH*; subarachnoid hemorrhage, *F*; female, *M*; male, *A-com A*; anterior communicating artery, *VA*; vertebral artery, *SCA*; superior cerebellar artery, *MCA*; middle cerebral artery.

## Conclusion

We described a case of SAH secondary to ruptured saccular intracranial aneurysm in a 37-year-old female patient with clinically diagnosed OI. She was successfully treated with the surgical neck clipping of the aneurysm. Although no mutation in the *COL1A1* or *COL1A2* gene was detected, it is obvious that this patient is clinically OI. There may be some causative relationship between OI and SAH because there are several reported cases of SAH secondary to cerebral aneurysm in patients with OI.

## Consent

We obtained written informed consent from the patient for publication of this case report and any accompanying images. A copy of the written consent is available for review by the Editor-in-Chief of this journal.

## Abbreviations

OI: Osteogenesis imperfecta; COL1A2: Collagen type 1 alpha-2 gene; IA: Intracranial aneurysm; SAH: Subarachnoid hemorrhage; DSA: Digital subtraction angiography; SNP: Single nucleotide polymorphism.

## Competing interests

We have no disclosures and did not receive any financial support.

## Authors’ contributions

TH participated in treatment of the patient, and drafted the all manuscript. SM, TI, SY, HN, YS, SM, KH and MM participated in treatment of the patient, and helped to draft the manuscript. AM performed the genetic analysis for a SNP of exon 28 of the COL1A2. AM conceived of the case study, and participated in its design and management. All authors read and approved the final manuscript.

## Authors’ information

AM is the professor of Department of Neurosurgery, Teikyo University, transferred from Teikyo University Chiba Medical Center in April, 2014.

## Pre-publication history

The pre-publication history for this paper can be accessed here:

http://www.biomedcentral.com/1471-2377/14/150/prepub
